# Prenatal Maternal Immunization for Infant Protection: A Review of the Vaccines Recommended, Infant Immunity and Future Research Directions

**DOI:** 10.3390/pathogens13030200

**Published:** 2024-02-23

**Authors:** Elizabeth M. Quincer, Lisa M. Cranmer, Satoshi Kamidani

**Affiliations:** 1 Immunization Safety Office, Centers for Disease Control and Prevention, Atlanta, GA 30333, USA; 2 Department of Pediatrics, Emory University School of Medicine, Atlanta, GA 30322, USA; 3 Children’s Healthcare of Atlanta, Atlanta, GA 30322, USA; 4 Department of Epidemiology, Emory University Rollins School of Public Health, Atlanta, GA 30322, USA

**Keywords:** maternal immunization, maternal child health, pediatric infectious diseases, pregnancy, immunology

## Abstract

Prenatal maternal immunization is an effective tool to protect mothers and infants from poor health outcomes due to infectious diseases. We provide an overview of the rationale for the use of prenatal vaccines, discuss the immunologic environment of the maternal–fetal interface including the impact of maternal vaccines prenatally and subsequently on the infant’s immune response, and review vaccines currently recommended in pregnancy and landscape for the future of maternal vaccination. This review aims to provide an understanding of the recent history and progress made in the field and highlight the importance of continued research and development into new vaccines for pregnant populations.

## 1. Background and Rationale

Globally, childhood mortality disproportionally occurs in the perinatal period, with over 15% of all neonatal deaths due to an infectious etiology [1]. Maternal infections are a driver of preterm birth, which also contributes to neonatal mortality [2]. During the critical period before infants have initiated their primary immunization series, maternal vaccination serves as a safe and effective tool to reduce perinatal morbidity and mortality from infectious diseases, including tetanus, pertussis, influenza, COVID-19 caused by severe acute respiratory syndrome coronavirus 2 (SARS-CoV-2), and respiratory syncytial virus (RSV) [3,4]. Maternal immunization has demonstrated substantial impact in preventing a range of early neonatal and childhood infections. Implementation of antenatal tetanus vaccination programs decreased global neonatal tetanus mortality by 92% [5], prenatal influenza immunization reduced infant respiratory illness by 50% in Bangladesh [6], and antenatal pertussis immunization protected 91% of infants <3 months of age during an outbreak in England [7]. Maternal COVID-19 vaccination protects infants less than 6 months of age from hospitalization for COVID-19 with a vaccine effectiveness of 52% for hospitalization and 70% for intensive care unit admission [8]. Recently, maternal bivalent RSV prefusion F protein-based (RSVpreF) vaccination (Pfizer, Abrysvo) was found to be 82% efficacious protecting against severe RSV infection in infants less than 3 months of age in clinical trials [9]. (Table 1) Further, maternal vaccination also has the potential to combat pregnancy complications, as influenza in pregnancy is associated with pregnancy loss and low birthweight [10], and COVID-19 disease in pregnancy can lead to stillbirth and preterm birth [11].

The field of maternal immunization has advanced considerably, and today, while each country has specific guidance for immunization recommendations in pregnancy and global coverage in pregnancy varies, the World Health Organization (WHO) recommends that pregnant women (We use the term ‘women’ throughout this review, which may include cis-gender females, transgender males, and non-binary individuals) and their newborns are protected from birth-associated tetanus, consideration for a pertussis-containing vaccine in pregnancy is given in countries with high infant morbidity or mortality from pertussis, seasonal influenza vaccine should be prioritized for pregnant women, and COVID-19 vaccination should be administered in pregnancy when the benefits outweigh the potential risks [51]. The United States (US) Advisory Committee on Immunization Practices (ACIP) recommends tetanus, pertussis, influenza, COVID-19, and RSV vaccines during pregnancy [52]. (Table 2) The history of maternal vaccination began in the 19th century with reports demonstrating protection against smallpox in pregnant women who had received smallpox vaccination prior to pregnancy compared to those who had not received vaccination [53]. Subsequent research in the 20th century focused on transplacental antibody transfer. Infants of women who had received smallpox vaccine in pregnancy were found to be refractory to subsequent immunization with the live attenuated smallpox vaccine strain after birth. Additionally, transplacental transfer of tetanus and diphtheria toxoid antibodies was demonstrated [53]. An inactivated influenza vaccine was licensed for the general population in the United States in 1945 [54] but was not recommended in pregnancy until 1960 (A paucity of active research on maternal immunization carried out during this time was due to product safety concerns in pregnancy, particularly after the experience with thalidomide in the 1960s, which was found to be associated with severe limb and other deformities in infants born to women who took this unlicensed medication to treat hyperemesis gravidarum) [13,55]. In 1974, the World Health Organization’s (WHO) Expanded Program on Immunization (EPI) made elimination of neonatal tetanus through maternal vaccination initiatives a priority [56]. This was further reinforced in 1999 by the World Health Assembly as the Maternal and Neonatal Tetanus Elimination (MNTE) initiative [5]. Following only modest impact from efforts to protect infants from pertussis through ‘cocooning’, or from the vaccination of adults and adolescents who have close contact with infants [57], pertussis vaccination in every pregnancy was recommended in the United States in 2012 [58]. 

Pregnant and lactating women have historically been excluded from vaccine clinical trials, which has limited evaluation of vaccine safety and effectiveness data in pregnant populations to post-licensure studies using both passive and active surveillance methods (e.g., the Vaccine Adverse Event Reporting System (VAERS), Vaccine Safety Datalink (VSD), V-safe, COVID-19 Vaccine Pregnancy Registry, US Flu Vaccine Effectiveness (VE) Network, Investigating Respiratory Viruses in the Acutely Ill Network, National Vaccine Surveillance Network (NVSN), and the VISION VE Network) [63,64,65,66]. The recognition early in the COVID-19 pandemic response that pregnancy was a high-risk condition led to an important historical precedent of including vaccination of pregnant women in phase 3 clinical trials (albeit post-initial emergency use authorization [67]). In addition, pregnant women were included early in vaccine rollout and, ultimately, in post-licensure surveillance studies [68,69]. This has paved the way for further research on immunizations targeting key pathogens for pregnant and neonatal populations. In 2023, a vaccine to protect infants from RSV was licensed and recommended for use in pregnant persons [50,70]. 

In this review, we discuss the maternal–fetal interface and its important immunologic properties in ensuring adaptation to the semi-allogenic fetus, sustaining fetal immune development, and maintaining defense against various infections, along with vaccines currently recommended for use during pregnancy and a new maternal vaccination for Group B Streptococcus (GBS) under development.

## 2. The Maternal–Fetal Interface and Fetal Immune Responsiveness

Alterations in the maternal immune system during pregnancy leave pregnant individuals at increased risk for infections. There is a complex interplay between changing levels of estrogen and progesterone during pregnancy, which are associated with a shift from Th1 to Th2 immune responses [71,72], while other components of the maternal immune system, such as phagocytic activity, alpha-defensin expression, and numbers of neutrophils, monocytes, and dendritic cells, are maintained or increased in pregnancy [73]. The immune alterations in pregnancy lead to increased susceptibility to such infectious diseases as influenza, listeriosis, toxoplasmosis, tuberculosis, human immunodeficiency virus (HIV), and malaria, among others [73]. The maternal–fetal interface is immunologically complex, including the maternal decidua and fetal villous chorion, which ensures immune tolerance towards the semi-allogenic fetus while providing immune defense against infection [74]. The placenta acts to restrict vertical transmission of pathogens during pregnancy through both structural and immune barriers; however, certain pathogens, such as those that cause congenital infections (‘TORCH’ or *Toxoplasma gondii*, rubella virus, cytomegalovirus (CMV), herpes simplex virus, and other viruses) have developed mechanisms for placental defense evasion [75]. 

The fetal immune system transitions from a state of immune tolerance in utero to a state of protective immunity that is needed at the time of birth [76]. T cells preferentially differentiate into regulatory T (T_reg_) cells during fetal growth, which are important for suppression of both the expansion and activation of effector T cells necessary for in utero survival of the semi-allogeneic fetus [76,77]. While the fetal immune system was historically deemed ‘immature’, with a bias towards a Th2 immune response [78] important for protecting the fetus from potentially harmful Th1-mediated inflammation, it has more recently been shown that T cells capable of a Th1 response are present in the fetus [79,80]. This fetal Th1 response may play an important role in protecting the fetus against in utero maternal infection with pathogens such as CMV and malaria, among others [81,82]. In late gestation and after birth, T_reg_ cells are downregulated, which is thought to be important for the development of neonatal immunity against infectious pathogens [77,83]. 

## 3. Influence of Antenatal Maternal Vaccination on Infant Immunity

Maternal vaccination elicits production of antigen-specific maternal antibodies, which are transferred across the placenta to the fetus and reduce the risk of disease and associated complications during infancy [84]. Maternal antibody transplacental transfer occurs via transcytosis mediated by the neonatal Fc receptor expressed on the surface of syncytio-trophoblasts, with IgG as the predominant isotype transferred [85]. Passive immunization through maternal antibody transfer provides the infant with a varied repertoire [86] of antigen-specific antibodies in the critical period following birth, with a half-life extending from 28 to 35 days [87]. Many factors impact the concentration of maternal antibodies transferred to the infant, including both antibody-specific factors, such as antibody isotype, subclass, and glycosylation profile, as well as the infant’s gestational age, birth weight, and the presence of maternal comorbidities [88].

Maternal vaccine-induced antibodies have been shown to interfere with subsequent antibody response in the infant to childhood immunizations, likely by inhibiting cross-linking of the B cell receptor and Fc receptor by vaccine antigen and maternal antibody, thereby inhibiting B cell proliferation and antibody production [89]. This ‘blunting effect’ on immunogenicity in infants following vaccination has been demonstrated for both maternal vaccine-targeted (pertussis [90,91] and diphtheria) and non-targeted (pneumococcus [92] and polio [93]) pathogens [94]. Acellular pertussis vaccine has demonstrated greater attenuation of infant immunogenicity as compared to receipt of whole-cell pertussis vaccine owing to greater induction of maternal antibodies by the former [95]. While a blunting effect was noted after two doses of pneumococcal vaccine among infants born to mothers who had received tetanus toxoid, reduced diphtheria toxoid, acellular pertussis (Tdap) vaccination during pregnancy, maternal Tdap vaccination had a minimal impact on overall pneumococcal sero-protection rates after infant primary and booster vaccination [92]. While lower infant antibody titers were observed following early infant measles vaccination in the presence of maternal measles antibodies, antibody titers more similar to those of infants who did not have maternally-acquired measles antibodies were achieved following revaccination [96,97]. Nonetheless, no clinically significant effects of maternal antibody interference on humoral and cellular immune responses to childhood immunizations have been shown to date [98,99]. 

Another area of study related to infant immune responses that remains an open question in some instances is the impact of co-administration of vaccines during pregnancy on maternal vaccine effectiveness. In a recent trial in non-pregnant women evaluating the safety and immunogenicity of co-administered Tdap and RSV vaccines, non-inferiority criteria for pertussis immune responses were not met [100]. Further research will be needed to elucidate if co-administration of antenatal Tdap and RSV might have any impact on young infants’ susceptibility to acquiring clinical pertussis infection.

Protective effects of maternal vaccination beyond antigen-specific passive humoral immunity have been demonstrated. Influenza vaccination during pregnancy has been shown to decrease all-cause lower respiratory tract infections during the first 3 months of an infant’s life, suggesting non-targeted protection against secondary bacterial infections to which influenza infection itself may predispose [101]. Similarly, maternal influenza vaccination given in combination with infant pneumococcal vaccination may also confer greater protection for acute respiratory infections compared to infant pneumococcal vaccination alone [102,103]. Non-specific decreases in infectious risk have also been shown in regard to maternal pre-pregnancy Bacille Calmette-Guérin (BCG) vaccination; in studies of infants who themselves received BCG vaccination, fewer hospitalizations for infections and lower mortality were noted in the setting of maternal BCG priming [104,105,106]. Additionally, and seemingly inconsistent with the impact of circulating maternal antibodies on the antibody levels achieved through early vaccination discussed above, a lower mortality rate was found in children living in low-income settings who received early measles vaccination in the presence of circulating maternal measles antibody compared to children vaccinated without pre-existing maternal measles antibody, even when controlling for potential factors such as birth weight, weight-for-age z-score, and breastfeeding, among others [107]. While the underlying biological mechanisms remain elusive, it is speculated that maternal measles antibodies may enhance cellular immune responses through increased presentation of antigen-maternal antibody complexes [107]. A study evaluating non-specific effects of routine childhood vaccination on infectious disease hospitalization in children 16–24 months of age found a decreased risk of non-targeted infectious disease hospitalization if the child had last received a live vaccine compared to an inactivated vaccine only [108]. In addition to antibodies, maternal cells are transferred to the fetus during pregnancy and have an impact on the developing fetal immune system [109]. For example, maternal micro-chimerism, the bidirectional transfer of genetically distinct cells between the mother and fetus, was associated with an improved infant polyfunctional CD4 response to BCG vaccination in South African infants [110]. Maternal vaccine antigens have been shown to prime the infant’s B and T cell immune responses [84,111,112]. 

Vaccines administered during pregnancy and postpartum also provide passive antibody transfer to the infant’s respiratory and gastrointestinal mucosa via breast milk, with IgA predominating over IgG and IgM [113]. Vaccine-specific antibodies are endocytosed into the mammary gland epithelial cells with subsequent secretion in colostrum and breast milk [114]. While mechanistic effects of breast milk antibodies on infant mucosal immunity are unclear, enhanced breast milk humoral immunity after maternal intramuscular influenza vaccination has been associated with lower rates of infant respiratory illness with fever [115]. The route of maternal immunization may also be important in shaping breast milk immunity, as maternal immunization with live attenuated influenza vaccination enhanced innate immune cellular responses in breast milk in addition to eliciting antigen-specific antibodies induced by parenteral vaccination [116].

## 4. Vaccines Recommended during Pregnancy in the United States

Recommended vaccines in pregnancy in the United States are detailed below and summarized in Table 1 and Table 2.

### 4.1. Influenza

Influenza infection in pregnancy is associated with an increased risk for hospitalization, severe illness, and poor pregnancy outcomes, and this has been shown for both pandemic H1N1 influenza [117,118] and annual seasonal influenza [119]. Neuzil et al. reported an estimated attributable risk (AR) of 1.91 (95% confidence interval (CI), 1.51–2.31) per 10,000 women-months for influenza-related cardiopulmonary hospitalizations among pregnant compared to non-pregnant women [12]. Influenza in pregnancy is also associated with late pregnancy loss (adjusted hazard ratio (aHR) 10.7, 95% CI, 4.3–27.0) and a reduction in mean birthweight (−55.3 g, 95% CI, −109.3–−1.4 g) [10]. Infants have among the highest rates of both influenza infection and influenza-associated hospitalization [13,14], and children under 6 months of age, for whom there is no currently recommended influenza vaccination, are at the highest risk for influenza-associated pediatric deaths [14,15]. Influenza vaccination is recommended during every pregnancy in the United States to protect pregnant women and to provide passive immunization to their infants during the first months of life [52], and the WHO Strategic Advisory Group of Experts notes that pregnant individuals should be prioritized for vaccination [120]. However, many countries still do not have recommendations for influenza vaccination in pregnancy [3]. Even in countries with influenza vaccine recommendations in pregnancy, much room for improvement in uptake remains. A recent survey in the United States found that 47.2% of women reported influenza vaccination prior to or during their pregnancy in the 2022–2023 influenza season [121].

In a series of four randomized controlled trials conducted in Bangladesh [18], Nepal [16], South Africa [19], and Mali [17], vaccine efficacy for maternal influenza immunization in mothers ranged from 31% to 70% and in their infants from 30% to 63% for influenza-like illness or laboratory confirmed influenza. Subsequent pooled analysis of the three randomized controlled trials conducted in Nepal, Mali, and South Africa revealed a pooled efficacy of maternal influenza vaccination in preventing polymerase chain reaction-confirmed influenza in infants up to 6 months of age at 35% (95% CI, 19–47%) [20]. This efficacy was highest within the first two months of life, at 56% (95% CI, 28–73%) [20]. Furthermore, comparable efficacy of influenza vaccination in pregnancy for infants less than 6 months of age was observed in high-resource settings, with a rate of 56.8% (95% CI, 25.0–75.1%) [122]. More recent data demonstrated vaccine effectiveness of 34% (95% CI, 12–50%) against influenza-related hospitalization and emergency department visits in infants less than 6 months of age and 53% (95% CI, 30–68%) in infant less than 3 months of age in a prospective, test-negative case control study from NVSN [123]. Studies have consistently shown that maternal influenza vaccination is safe for the mother and not associated with any increased risk of pregnancy or neonatal adverse outcomes; these include conditions such as chorioamnionitis, preeclampsia, eclampsia, birth defects, preterm birth, and low birthweight [20,21,22,23,24]. An early study in the VSD found no association between spontaneous abortion and influenza vaccination [28]. A subsequent VSD study following the H1N1 pandemic found an association between spontaneous abortion and influenza vaccination in the preceding 28 days with effect modification among women who had received pH1N1-containing influenza vaccine in the prior season [26]. However, this association was not replicated in a larger VSD study, including women vaccinated in a preceding influenza season, further supporting the safety of maternal influenza vaccination [27]. There has been no observed association between maternal influenza vaccination and adverse health outcomes in early childhood, including immune-related (e.g., asthma, infections) and non-immune-related health outcomes (e.g., neoplasms) [25].

### 4.2. Tetanus, Diphtheria and Pertussis

Universal maternal tetanus toxoid vaccination is recommended for all pregnant women to prevent neonatal mortality from tetanus [124]. Prior to routine maternal immunization, tetanus worldwide was reported to cause 15,000–30,000 maternal deaths per year and account for approximately 5% of all maternal mortality [29]. Neonatal tetanus contributed significantly to neonatal mortality with an 80–100% case-fatality rate in low-resource settings [124]. The World Health Assembly recommended elimination of neonatal tetanus in 1989, which was reinforced in 1999 as the MNTE initiative; as a result, all but 12 countries have implemented MNTE leading to an 88% reduction in neonatal tetanus cases and a 92% reduction in mortality due to neonatal tetanus worldwide [5]. Widespread use of tetanus toxoid vaccines in pregnant women has not led to any safety concerns, including no evidence for increased risk of pregnancy complications, local or systemic side effects and no safety concerns for the infant, including no evidence for congenital anomalies [30,31]. Additionally, no safety concerns were demonstrated following Tdap administration in pregnant women who had prior receipt of tetanus-containing vaccines [31]. Few studies have examined the effect of Tdap vaccination during pregnancy for neonatal protection against diphtheria. One study found protection against diphtheria, defined as anti-diphtheria IgG ≥ 0.1 IU/mL, in 100% of infants born to women who received Tdap vaccination during pregnancy compared to 62.5% of infants whose mothers had received Tdap prior to pregnancy, providing further support for Tdap vaccination in pregnancy [125].

Tdap vaccination of pregnant women is indicated to prevent pertussis in young infants and has been recommended since 2012 in the United States for use in every pregnancy [58]. Tdap vaccination is recommended in pregnancy in several high-income countries, but recommendations in many low-and middle-income countries are lacking; further, many countries that do recommend Tdap vaccination in pregnancy do not consistently have high vaccination coverage [126]. A survey among women in the United States who had a recent live birth found 55.4% reported Tdap vaccination coverage during pregnancy [121]. Recommendations for Tdap vaccination in pregnancy have arisen in response to large pertussis outbreaks; as such, most of the safety and effectiveness data come from associated observational studies. During a pertussis outbreak in England, prenatal maternal Tdap immunization was associated with vaccine effectiveness of 93% (95% CI, 81–97%) and 91% (95% CI, 84–95%) [7] in reduction of laboratory-confirmed cases of pertussis in infants <2 months of age and <3 months of age, respectively [127]. In the United States, prenatal maternal Tdap vaccination was associated with 91% (95% CI, 20–99%) vaccine effectiveness during the first 2 months of an infant’s life and 69% (95% CI, 44–83%) vaccine effectiveness during the entire first year of an infant’s life [34]. A systematic review confirmed these estimates of high effectiveness, ranging from 69–93% among infants aged less than 3 months following second or third trimester maternal vaccination across the included observational studies [128].

While one study from two VSD sites suggested a slight increase in chorioamnionitis among Tdap-vaccinated pregnant women [129], this finding was not corroborated in a subsequent VSD study involving over 100,000 pregnancies across eight VSD sites [35]. The latter study employed more rigorous statistical analyses to address specifically the immortal time bias that was not considered in the prior study [35,129]. Furthermore, the study demonstrated low positive predictive value in the 10th revision of the International Statistical Classification of Disease (ICD-10) coding used for the diagnosis of chorioamnionitis (48% for clinical and 59% for histologic chorioamnionitis), underscoring the importance of conducting chart reviews to avoid outcome misclassification [35]. The absence of an association between receipt of the Tdap vaccine during pregnancy and chorioamnionitis is also consistent with the findings of other studies [36,37]. Data on the safety of maternal Tdap vaccination have otherwise been reassuring, with no identified risk for severe maternal adverse events or adverse pregnancy and neonatal outcomes reported [38,39]. The safety of repeat Tdap vaccination was studied in the VSD in non-pregnant female and male participants and no increased risk of adverse medical outcomes, including seizure, cranial nerve disorders, limb swelling, pain in limb, cellulitis, paralytic syndromes and encephalopathy, encephalitis, or meningitis with repeat Tdap vaccination, was found [130].

### 4.3. COVID-19

Pregnant women are at increased risk for severe illness due to COVID-19 compared to non-pregnant women, including the need for intensive care (odds ratio (OR) 2.61, 95% CI, 1.84–3.71), the requirement for mechanical ventilation (OR 2.41, 95% CI, 2.13–2.71) and risk of maternal death (OR 6.09, 95% CI, 1.82–20.38) [11]. They are also more susceptible to adverse pregnancy and neonatal outcomes compared to pregnant women without COVID-19 such as stillbirth (OR 1.81, 95% CI, 1.38–2.37), preterm birth (OR 1.57, 95% CI, 1.36–1.81), and their infant’s needing admission to neonatal intensive care (OR 2.18, 95% CI, 1.46–3.26) [11]. These adverse outcomes were further exemplified during the Delta period with an increase in stillbirths (0.7% versus 0.4%, adjusted prevalence ratio (aPR) 1.55, 95% CI 1.14–2.09) and preterm births (12.8% versus 11.9%, aPR 1.14, 95% CI 1.07–1.20) compared to the pre-Delta period [131]. While pregnant women were excluded from pre-licensure vaccine clinical trials, they were offered the opportunity to receive the vaccine during the initial rollout of COVID-19 vaccination in December 2020 [69]. This decision was based on the premise that the potential benefits of vaccination outweighed the risks. Data from early after COVID-19 vaccination rollout from a study in Israel showed the estimated vaccine effectiveness of two doses of Pfizer–BioNTech’s BNT162b2 vaccine for laboratory-confirmed SARS-CoV-2 infection in pregnant women to be 78% (95% CI, 57–89%) [40]. Similarly, a study from Qatar showed that vaccine effectiveness for two doses of either available mRNA vaccine (Pfizer–BioNTech’s BNT162b2 and Moderna’s mRNA-1273) from 30 December 2020 to 30 May 2021 was 88% (95% CI, 44–97%) using a cohort analysis [41]. Maternal receipt of the two dose-series of COVID-19 mRNA vaccines during pregnancy has also been shown to protect infants six months of age and younger from hospitalization for COVID-19, with a vaccine effectiveness of 52% (95% CI, 33–65%) for hospitalization overall, 80% (95% CI, 60–90%) during the Delta period and 38% (95% CI, 8–58%) during the Omicron period [8]. Many studies have evaluated the safety of COVID-19 vaccination during pregnancy since the vaccine rollout and have not found any risk for adverse events, including local or systemic maternal effects, and pregnancy and neonatal conditions, including spontaneous abortion, stillbirth, preterm birth, small for gestational age and infant hospitalization [4,42,43,44,45,46]. A decreased risk of stillbirth in those who received COVID-19 vaccination (primarily, but not limited to, mRNA COVID-19 vaccination across studies) during pregnancy has been shown in multiple studies [132,133].

Despite the established efficacy and safety of COVID-19 vaccines, vaccine coverage among pregnant women remains suboptimal, with a recent survey of pregnant US women reporting that 64.9% received ≥1 COVID-19 vaccine dose, 58.7% completed the primary COVID-19 vaccination series, and 27.3% received a bivalent COVID-19 booster dose [121,134,135]. An increasing trend in COVID-19 vaccine hesitancy has been observed among pregnant women [136]. COVID-19 vaccine hesitancy during pregnancy in the United States has been found to be associated with race, ethnicity and socio-economic status, with lower uptake among Hispanic and non-Hispanic Black women [135] and in those living in areas with higher social vulnerability [137]. Given this vaccine hesitancy, especially among populations who may be at higher risk for adverse outcomes due to COVID-19, the US Centers for Disease Control and Prevention (CDC) and professional medical organizations have reinforced the importance of COVID-19 vaccination in pregnancy and the need to leverage efforts to increase equitable vaccine uptake [138]. Furthermore, with the demonstrated waning of COVID-19 vaccine effectiveness [139] and the development of new COVID-19 variants [140], it is increasingly important to ensure pregnant women are up-to-date with COVID-19 vaccination.

### 4.4. Respiratory Syncytial Virus

Respiratory Syncytial Virus (RSV) is a leading cause of lower respiratory tract infection (LRTI) in infants with an estimated 68% of infants infected during the first year of life and nearly all infants (97%) infected by two years of age [47]. It is estimated that 2–3% of all infants are hospitalized for RSV infection [48]. While premature infants have a higher risk of hospitalization, 79% of children under the age of two years hospitalized for RSV have no underlying medical condition [49]. In the 1960s, a formalin-inactivated, alum-precipitated, whole-virus RSV vaccine administered to infants was found to cause potentially life-threatening vaccine-associated enhanced respiratory disease, a phenomenon attributed to the production of non-neutralizing antibodies and a Th2-biased immune response [141,142,143]. This vaccine safety concern significantly hindered subsequent RSV vaccine development. However, innovative strategies have since been devised to circumvent or minimize this potential adverse reaction. Two vaccine candidates from Pfizer (Abrysvo) and GSK (Arexvy) were pursued, which utilize an RSV F protein stabilized in the prefusion conformation and which were shown to generate durable neutralizing antibody and cell-mediated immune responses [144,145]. These RSV vaccines were shown to protect against RSV-associated acute respiratory illness in adults ≥60 years of age in phase 3 clinical trials [146,147]. In May 2023, the US Food and Drug Administration (FDA) approved and, in June 2023, ACIP recommended that persons aged ≥60 years could receive these vaccines, using shared clinical decision-making, to prevent symptomatic RSV-associated lower respiratory tract disease [148]. 

A phase 3 clinical trial of Pfizer’s bivalent RSVpreF protein-based vaccine (Abrysvo) administered to pregnant women at 24 to 36 weeks gestation showed vaccine efficacy for medically-attended severe RSV-associated LRTI within 90 days after birth at 81.8% (99.5% CI, 40.6–96.3%) and for medically attended RSV-associated LRTI within 90 days of birth at 57.1% (99.5% CI, 14.7–79.8%) [9]. No statistically significant safety signals were detected in maternal participants or in infants and toddlers up to 24 months of age. However, a non-statistically significant numerical imbalance in preterm delivery was observed: 5.7% (95% CI, 4.9–6.5%) in the vaccine group compared to 4.7% (95% CI, 4.1–5.5%) in the placebo group [9,149]. Limiting the dosing interval, ACIP judged the benefit of maternal RSVpreF vaccination at 32 to 36 weeks in preventing RSV-associated LRTI in infants to outweigh the risks, including the potential risks for preterm birth and hypertensive disorders of pregnancy [50]. Of note, GSK halted its phase 3 clinical trials for a similar maternal RSV vaccine product (Arexvy) due to an increase in preterm delivery and consequent neonatal death in the vaccinated group [150,151]. Neuroinflammatory conditions were reported as an adverse safety outcome in Pfizer’s bivalent RSV vaccine (Abrysvo) phase 3 trial in older adults [146]. While this safety outcome was not found in pregnant persons administered RSVpreF, it will be important for ongoing safety monitoring to evaluate for any adverse neuroinflammatory conditions after RSV vaccination in pregnancy. 

In May 2023, the US FDA Vaccines and Related Biological Products Advisory Committee voted in support of Pfizer’s RSVpreF vaccine candidate (Abrysvo) for use in pregnant persons to combat RSV in infants [152], and FDA licensed its use in August 2023 [70]. ACIP and CDC subsequently made recommendations for the use of RSVpreF for pregnant persons at 32–36 weeks’ gestation as a seasonal vaccination for protection against RSV-associated LRTI in infants under 6 months of age [50]. FDA and ACIP limited the dosing interval to 32–36 weeks’ gestation to avoid potential risk for preterm birth at <32 weeks’ gestation [50,70]. In contrast, the GSK vaccine (Arexvy) is not approved nor recommended for use in pregnant persons. Nirsevimab, a long-acting monoclonal antibody, was also licensed and recommended for use in infants in their first RSV season to prevent RSV-associated LRTI (certain high-risk infants are also recommended to receive nirsevimab in their second RSV season). All infants are recommended to be protected against RSV through either maternal vaccination or nirsevimab administration, but most infants do not need protection from both products [50]. Ongoing post-licensure monitoring will evaluate vaccine safety in real-world applications.

## 5. Landscape for the Future of Maternal Immunizations

New maternal vaccines are currently in various stages of research and development, aiming to target diseases that pose significant risks to pregnant women and their infants. 

### 5.1. Group B Streptococcus

GBS maternal infection is associated with adverse birth outcomes, including preterm birth and stillbirth [153], as well as early and late-onset disease in infants manifesting as sepsis, pneumonia, or meningitis [154]. Universal maternal screening for GBS and intrapartum antibiotic prophylaxis are recommended for prevention of GBS disease; however, these recommendations have only resulted in a reduction in early-onset GBS disease and have not been effective in preventing late-onset disease or birth outcomes [153]. In addition, implementing such interventions may not be feasible in resource-limited countries or settings while a significant worldwide burden from GBS disease remains [155]. To prevent GBS disease in infants, efforts to develop a vaccine targeting GBS during pregnancy initially utilized a monovalent vaccine approach [156]. Due to the lack of cross protection against other serotypes, subsequent attempts have used a trivalent conjugate vaccine targeting capsular serotypes Ia, Ib, and III, which were found to be safe and immunogenic in early clinical trials [157,158,159]. However, worldwide serotype distribution also includes serotypes II, IV and V, all together (Ia, Ib, and II through V) accounting for 98% of identified colonizing GBS isolates [160]. Recently, a hexavalent glycoconjugate vaccine has been developed, and vaccination in pregnant women has demonstrated the production of antibodies that were transferred to infants at protective levels against invasive GBS disease [161]. Such sero-epidemiologic studies aiming to establish sero-correlates of protection are of paramount importance, especially since conducting phase 3 trials might not be feasible due to the relatively low incidence of GBS disease [162]. Moreover, a novel approach for developing a vaccine candidate to target the GBS alpha-like surface protein is currently in development [163]. A recent study estimated that worldwide GBS maternal vaccination could prevent 127,000 infant and maternal GBS cases, 23,000 stillbirths, and 37,000 infant deaths, given a vaccine efficacy of 80% and 50% coverage [164].

### 5.2. Future Research Directions

While hexavalent glycoconjugate GBS vaccine is potentially the leading candidate in development for use in pregnancy, several other pathogens are also being targeted for future maternal immunization, including zika virus, CMV, HIV, malaria, and extra-intestinal *Escherichia coli,* among others. Congenital CMV remains a significant contributor to neonatal morbidity, particularly due to sensorineural hearing loss. The incomplete understanding of the immune correlates of protection for the fetus has limited progress in the development of a maternal vaccine, indicating a clear need for further research [165]. Despite these challenges, significant and continuous efforts have been dedicated to the development of a CMV vaccine, exploring various vaccine platforms, including attenuated vaccines, vectored vaccines, and both DNA and mRNA vaccines [166]. Malaria infection in pregnancy, especially when primigravid, can lead to significant morphological and immunological changes in the placenta and severe outcomes for the mother and infant, making development of a vaccine for malaria a high priority [167]. Vaccines targeting the protein expressed by malaria-infected erythrocytes which interacts with placental receptors are under development [167]. 

As the landscape of maternal immunization expands, it is essential for physicians, public health professionals, and academicians to collaborate on research and development. Areas for future research include studies to gain a deeper understanding of the maternal–fetal interface and its role in shaping infant immunity, which can help guide the development of more effective vaccination strategies. Investigating novel delivery methods and immunization schedules for administration of maternal vaccines in a variety of settings can contribute to enhancing their safety and efficacy. This may lead to better protection for both the mother and infant, ultimately resulting in improved maternal and neonatal health outcomes. It is critical now to strengthen the platform for delivering maternal immunizations globally, especially in low- and middle- income countries, given the impact these pathogens have on childhood mortality in such settings [1]. Further, many of these countries have not yet implemented routine Tdap and influenza vaccination in pregnancy and may not be well positioned to integrate additional novel maternal vaccinations when they are available. Finally, increased public awareness and education on the importance of maternal vaccination can encourage more widespread adoption of these life-saving interventions to maximize maternal immunization for infant protection on a global scale, especially considering the recent increase in vaccine hesitancy, which threatens to hamper progress in the field.

## 6. Conclusions

Maternal vaccination plays a crucial role in safeguarding the health of pregnant individuals and their infants, through the unique physiology of the maternal–fetal interface and its immunologic properties benefitting the developing fetus. The currently recommended maternal vaccines, including influenza, Tdap, and COVID-19 vaccines, have significantly reduced the incidence of disease and associated complications in these vulnerable populations. Further research is needed to understand the impact of maternal RSV vaccination in the setting of its recent licensure and recommendation for use in pregnancy. As the field continues to evolve, new maternal vaccines should be pursued to target other infectious diseases which contribute to global morbidity and mortality in pregnant women and their infants.

## Figures and Tables

**Table 1:** Summary of disease morbidity and mortality, vaccine efficacy or effectiveness, and safety of the currently recommended maternal vaccines in the United States.

Disease	Morbidity and Mortality	Vaccine Efficacy or Effectiveness	Vaccine Safety
Influenza	Maternal: increased risk of hospitalization, complications (AR 1.91, 95% CI, 1.51–2.31 per 10,000 women-months of influenza-related morbidity in pregnancy) [12] and poor birth outcomes from influenza in pregnancy including late pregnancy loss (aHR 10.7, 95% CI, 4.3–27.0) and reduction in mean birthweight (−55.3 g, 95% CI, −109.3–−1.4 g) [10]. Infant: high rates of influenza infection, hospitalization (125–375 per 100,000 persons) and mortality (0.73 per 100,000 persons) in infants < 6 months of age [13,14,15].	Maternal influenza vaccine efficacy against influenza-like illness or laboratory confirmed influenza: 31–70% for mothers and 30–63% for infants [16,17,18,19]. Pooled efficacy of maternal vaccination in preventing PCR-confirmed influenza in infants up to 6 months of age: 35% (95% CI, 19–47%) [20].	The body of evidence on the safety of maternal influenza vaccination does not suggest an increased risk for acute maternal, pregnancy, or neonatal adverse outcomes, and immune- and non-immune-related health outcomes and healthcare utilization in early childhood [20,21,22,23,24,25,26,27,28].
Tetanus	Maternal: prior to routine immunization, maternal tetanus was reported to cause 15,000–30,000 maternal deaths per year globally. Infant: neonatal tetanus can have a case fatality rate of 80–100% in resource-limited settings [29].	Impact of maternal tetanus vaccination on neonatal tetanus: 88% reduction in cases and 92% reduction in mortality worldwide [5].	No evidence for increased risk of pregnancy complications, local or systemic side effects, and no impact on birth or infant outcomes such as congenital anomalies including among women who received prior tetanus-containing vaccines [30,31].
Pertussis	Infant: greatest pertussis disease burden among infants, who are also at risk of greater morbidity and mortality due to pertussis. Infants ≤ 2 months of age have higher mortality compared to other infants (RR 2.6, 95% CI, 1.5–4.5) [32]. Infant: prior to immunization, pertussis incidence was 165.3 per 100,000 infants younger than 2 months of age in the United States [33].	Tdap vaccine effectiveness in pregnancy against pertussis: 91% (95% CI, 20–99%) in newborns <8 weeks of age and 69% (95% CI, 44–83%) in infants < one year of age [34].	No evidence for increased risk of chorioamnionitis [35,36,37] or additional safety concerns including adverse maternal, pregnancy, or neonatal outcomes following Tdap in pregnancy [38,39].
COVID-19	Maternal: increased COVID-19 severity of illness, with critical care admission (OR 2.61, 95% CI, 1.84–3.71) and respiratory support (OR 2.41, 95% CI, 2.13–2.71) in pregnant compared to non-pregnant women [11]. Maternal and Infant: maternal death (OR 6.09, 95% CI, 1.82–20.38), poor pregnancy outcomes including stillbirth (OR 1.81, 95% CI, 1.38–2.37), preterm birth (OR 1.57, 95% CI, 1.36–1.81) and neonatal intensive care unit admission (OR 2.18, 95% CI, 1.46–3.26) higher in pregnant women with versus without COVID-19 [11].	Vaccine effectiveness early in the COVID-19 pandemic: 78% (95% CI, 57–89%) [40] in Israel (Pfizer’s BNT162b2), 87.6% (95% CI, 44.1–97.2%) in Qatar (mRNA COVID-19 vaccine) for mothers [41]. 52% (95% CI, 33–65%) vaccine effectiveness against hospitalization in infants <6 months of age [8].	No pregnancy-specific safety concerns identified, including no adverse maternal acute and systemic reactions, pregnancy and neonatal outcomes, including spontaneous abortion, stillbirth, preterm birth, small for gestational age, and infant hospitalization [4,42,43,44,45,46].
RSV	Infant: 68% and 97% of infants estimated to be infected with RSV by one and two years of age, respectively [47]. Infant: hospitalization for RSV-associated LRTI occurs in 2–3% of all infants [48] with higher risk in premature infants, but 79% of those hospitalized have no underlying medical condition [49].	Pfizer’s bivalent RSVpreF vaccine (Abrysvo) administered at 24 to 36 weeks gestation showed 81.8% (99.5% CI, 40.6–96.3%) vaccine efficacy against severe RSV-associated LRTI in infants within 90 days of life [9]. Ongoing monitoring will inform vaccine effectiveness in real-world settings.	Numerical imbalance in preterm delivery was observed in Pfizer’s RSVpreF vaccine (Abrysvo) clinical trial with 5.7% (95% CI, 4.9–6.5%) in the vaccinated compared to 4.7% (95% CI, 4.1–5.5%) in the placebo group [9,50] *. No additional safety concerns in maternal or infant participants were identified [9]. Ongoing safety surveillance is needed for continued monitoring of preterm birth and other priority safety outcomes.

AR = attributable risk; CI = confidence interval; aHR = adjusted hazard ratio; PCR = polymerase chain reaction; RR = risk ratio; Tdap = tetanus, diphtheria, acellular pertussis; COVID-19 = Coronavirus disease 2019; OR = odds ratio; RSV = respiratory syncytial virus; LRTI = lower respiratory tract infection; RSVpreF = RSV prefusion F protein; * These estimates are for the Pfizer RSVPreF vaccine (Abrysvo) clinical trial which used a dosing interval of 24–36 weeks [9]. Advisory Committee on Immunization Practices (ACIP) recommendations limited the dosing interval to 32–36 weeks’ gestation to avoid potential risk for preterm birth at <32 weeks’ gestation. ACIP judged the benefit of vaccination at 32–36 weeks’ gestation in preventing RSV-associated LRTI in infants outweighed the risks, including the potential for preterm birth [50].

**Table 2:** Summary of vaccine recommendations in pregnant and breastfeeding persons for infant protection from the Advisory Committee on Immunization Practices in the United States [52,59].

	Advisory Committee on Immunization Practices Guidance
Influenza [60]	All persons aged ≥6 months, including those who are pregnant or who might be pregnant during the influenza season, should receive influenza vaccine. Any age appropriate IIV4 or RIV4 may be given in any trimester. LAIV4 should not be used during pregnancy but can be used postpartum.
Tetanus and Pertussis [61]	Pregnant persons who are not immunized or only partially immunized against tetanus should complete the primary series. A dose of Tdap should be administered during each pregnancy, irrespective of the patient’s prior history of receiving Tdap. Optimal timing for Tdap administration is between 27 and 36 weeks of gestation, although Tdap may be given at any time during pregnancy.
COVID-19 [62]	COVID-19 vaccination is recommended for all persons aged ≥6 months, including those who are pregnant, might become pregnant, were recently pregnant or are breastfeeding. The safety and effectiveness of COVID-19 vaccination indicates that the benefits of vaccination outweigh any potential risk of COVID-19 vaccination during pregnancy.
RSV [50]	Maternal RSV vaccine is recommended for pregnant people 32 through 36 weeks gestation to prevent LRTI in infants. Either maternal RSV vaccination during pregnancy at 32 through 36 weeks’ gestation or nirsevimab immunization for infants aged < 8 months who are born during or entering their first RSV season is recommended to prevent RSV-associated LRTI in infants, but administration of both products is not needed for most infants.

IIV4 = inactivated influenza vaccine, quadrivalent; RIV4 = recombinant influenza vaccine, quadrivalent; LAIV4 = live attenuated influenza vaccine, quadrivalent; Tdap = Tetanus, Diphtheria, and Pertussis vaccine; COVID-19 = Coronavirus disease 2019; RSV = respiratory syncytial virus; LRTI = lower respiratory tract infection.

## Data Availability

No new data were created or analyzed in this study. Data sharing is not applicable to this article.
